# (1-Methyl-1*H*-imidazole-κ*N* ^3^)(1-methyl-2-nitroso­benzene-κ*N*)(5,10,15,20-tetra­phenyl­porphyrinato-κ^4^ *N*)iron(II) di­chloro­methane monosolvate

**DOI:** 10.1107/S160053681400083X

**Published:** 2014-01-18

**Authors:** Erwin G. Abucayon, Dennis Awasabisah, Douglas R. Powell, George B. Richter-Addo

**Affiliations:** a Department of Chemistry and Biochemistry, University of Oklahoma, 101 Stephenson Pkwy, Norman, OK 73019, USA

## Abstract

The solvated title compound, [Fe(C_44_H_28_N_4_)(C_4_H_6_N_2_)(C_7_H_7_NO)]·CH_2_Cl_2_, is a porphyrin complex containing an octahedrally coordinated Fe^II^ atom with 1-methylimidazole [Fe—N = 2.0651 (17) Å] and *o*-nitro­sotoluene ligands at the axial positions. The *o*-nitro­sotoluene ligand is N-bound to iron(II) [Fe—N = 1.8406 (18)Å and Fe—N—O = 122.54 (14)°]. The axial N—Fe—N linkage is almost linear, with a bond angle of 177.15 (7)°. One phenyl group of the porphyrin ligand is disordered over two orientations in a 0.710 (3):0.290 (3) ratio. The di­chloro­methane solvent mol­ecule was severely disordered and its contribution to the scattering was removed with the SQUEEZE routine [van der Sluis & Spek (1990 ▶). *Acta Cryst.* A**46**, 194–201].

## Related literature   

Nitroso compounds are known to bind the Fe centers of many heme proteins including the blood protein hemoglobin (Keilin & Hartree, 1943 ▶; Hirota & Itano, 1978 ▶; Murayama, 1960 ▶; Gibson, 1960 ▶; Yi *et al.*, 2013 ▶). For the syntheses and crystal structures of related compounds, see: Wang *et al.* (1996 ▶); Godbout *et al.* (1999 ▶); Sohl *et al.* (2004 ▶). For a review on the inter­actions of *C*-nitroso compounds with metalloporphyrins, see: Lee *et al.* (2002 ▶). For the preparation of (TPP)FeCl (TPPH_2_ is 5,10,15,20-tetra­phenyl­porphyrin), see: Adler *et al.* (1970 ▶). For the use of SQUEEZE, see: van der Sluis & Spek (1990 ▶).
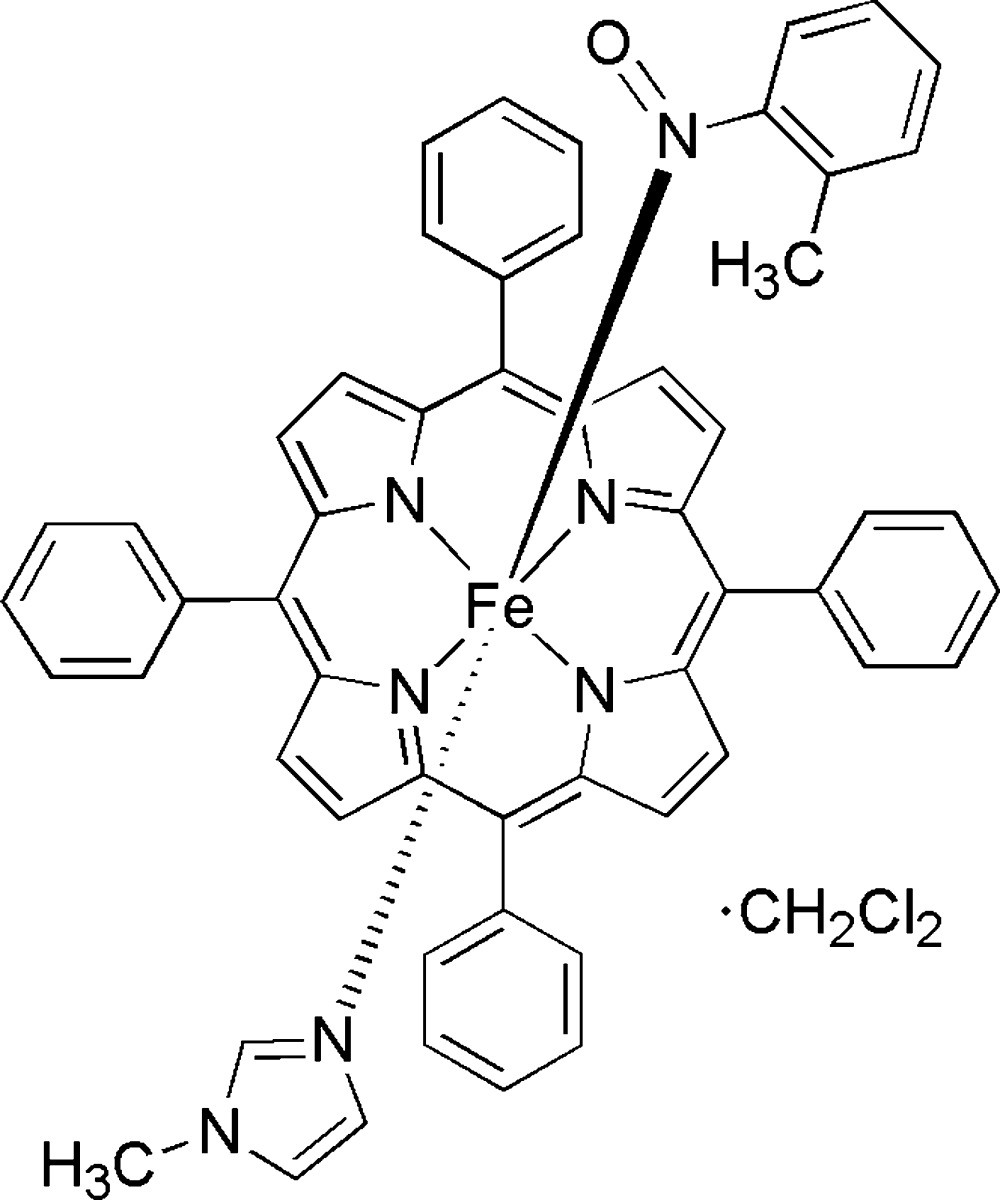


## Experimental   

### 

#### Crystal data  

[Fe(C_44_H_28_N_4_)(C_4_H_6_N_2_)(C_7_H_7_NO)]·CH_2_Cl_2_
*M* *_r_* = 956.72
Triclinic, 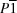

*a* = 12.1749 (12) Å
*b* = 13.4571 (13) Å
*c* = 15.0439 (15) Å
α = 107.450 (2)°
β = 94.800 (2)°
γ = 90.987 (2)°
*V* = 2340.8 (4) Å^3^
*Z* = 2
Mo *K*α radiation
μ = 0.49 mm^−1^
*T* = 100 K
0.54 × 0.15 × 0.08 mm

#### Data collection  

Bruker APEX CCD diffractometer
Absorption correction: multi-scan (*SADABS*; Bruker, 2002 ▶) *T*_min_ = 0.779, *T*_max_ = 0.962
43417 measured reflections
11596 independent reflections
8649 reflections with *I* > 2σ(*I*)
*R*_int_ = 0.044

#### Refinement  

*R*[*F* ^2^ > 2σ(*F* ^2^)] = 0.056
*wR*(*F* ^2^) = 0.173
*S* = 1.00
11596 reflections
632 parameters
353 restraints
H-atom parameters constrained
Δρ_max_ = 1.12 e Å^−3^
Δρ_min_ = −0.44 e Å^−3^

### 

Data collection: *SMART* (Bruker, 2007 ▶); cell refinement: *SAINT* (Bruker, 2007 ▶); data reduction: *SAINT*; program(s) used to solve structure: *SHELXS2013* (Sheldrick, 2008 ▶); program(s) used to refine structure: *SHELXL2013*; molecular graphics: *SHELXL2013*; software used to prepare material for publication: *SHELXL2013*.

## Supplementary Material

Crystal structure: contains datablock(s) I, New_Global_Publ_Block. DOI: 10.1107/S160053681400083X/hb7178sup1.cif

Structure factors: contains datablock(s) I. DOI: 10.1107/S160053681400083X/hb7178Isup2.hkl

CCDC reference:

Additional supporting information:  crystallographic information; 3D view; checkCIF report

## Figures and Tables

**Table 1:** Selected bond lengths (Å)

Fe1—N7	1.8406 (18)
Fe1—N1	1.9992 (17)
Fe1—N4	2.0030 (17)
Fe1—N2	2.0105 (16)
Fe1—N3	2.0159 (16)
Fe1—N5	2.0651 (17)

**Fig. 1.**
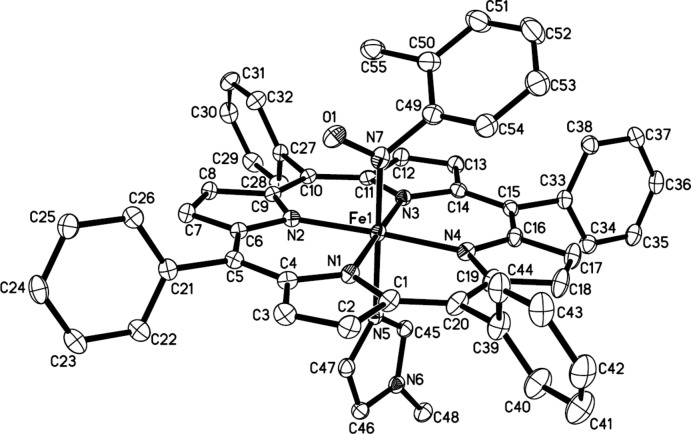
The molecular structure of (TPP)Fe(o-tolNO)(1-MeIm) with displacement ellipsoids drawn at the 50% probability level. H atoms and the dichloromethane solvent molecule are omitted for clarity.

## supplementary crystallographic information

### 1. Comment

Nitroso compounds are known to bind the Fe centers of many heme proteins including the blood protein hemoglobin (Keilin & Hartree, 1943; Hirota & Itano, 1978; Murayama, 1960; Gibson, 1960; Yi *et al.*, 2013). The synthetic bis-nitrosoarene iron porphyrin, (TPP)Fe(PhNO)_2_, was prepared and structurally characterized by X-ray crystallography (Wang *et al.*, 1996). We report the crystal structure of the six-coordinate (1-methyl-1*H*-imidazole-κ*N*^3^)(1-methyl-2-nitrosobenzene-κ*N*)(5,10,15,20-tetraphenylporphyrinato-κ^4^*N*)iron(II) dichloromethane monosolvate compound, (TPP)Fe(*o*-tolNO)(1-MeIm), (I). For the syntheses and crystal structures of related compounds, see: Wang *et al.* (1996); Godbout *et al.* (1999); Sohl *et al.* (2004). For a review, see: Lee *et al.* (2002). The molecular structure of (I) is shown in Fig. 1. The Fe—N(por) bond lengths are in the 2.0159 (16)–1.9992 (17) Å range. The Fe—N(1-MeIm) and Fe—N(*o*-tolNO) bond lengths are 2.0651 (17) and 1.8406 (18) Å, respectively. The axial N—Fe—N linkage shows a near linear geometry with a bond angle of 177.15 (7)°.

### 2. Experimental

To a Schlenk tube equipped with a magnetic stirrer was added (TPP)FeCl (61 mg, 0.09 mmol) and THF (15 ml). Zn(Hg) (60 mg, 0.9 mmol in Zn) was added and the mixture stirred for 2 h. The resulting solution was filtered into a clean Schlenk tube and the THF removed under vacuum and the residue subsequently dried. The purple (TPP)Fe^II^ solid obtained was dissolved in 15 ml of CH_2_Cl_2_, treated with 2-nitrosotoluene (50 mg, 0.4 mmol), and stirred for 12 h. The solution was reduced to 5 ml, and 10 ml hexane was added. The resulting solid was collected by filtration. The IR (KBr) spectrum showed the ν_NO_ band at 1350 cm^-1^, comparable with the 1353 cm^-1^ν_NO_ band observed for (TPP)Fe(PhNO)_2_ (Wang *et al.*, 1996). The (TPP)Fe(*o*-tolNO)_2_ obtained was then treated with 0.5 equivalent of 1-methylimidazole (purchased from Aldrich Chemical Company and used as received) and stirred for 30 min during which time the color of the solution changed from red-purple to brown-green. The solution was filtered and dried in vacuo. The IR (KBr) spectrum of the (TPP)Fe(*o*-tolNO)(1-MeIm) product showed the ν_NO_ band at 1366 cm^-1^. A suitable purple-needle shaped crystal was grown by slow evaporation of a CH_2_Cl_2_-hexane (1:1) solution of the complex at room temperature under N_2_.

### 3. Refinement

H atoms were located geometrically and treated as riding on their parent atoms with C–H = 0.95 Å for aromatic and 0.98 Å for methyl, and with *U*_iso_(H) = 1.2 (1.5 for methyl) times *U*_eq_(C). Restraints on the 1–2 (e.s.d. = 0.004Å) and 1–3 (e.s.d. = 0.008Å) contacts as well as planarity (e.s.d = 0.008Å) of the disordered phenyl groups were required. The displacement parameters of the disordered atoms were restrained to have similar values along bonded connections (e.s.d. = 0.003Å^2^).

### Crystal data

**Table:**

[Fe(C_44_H_28_N_4_)(C_4_H_6_N_2_)(C_7_H_7_NO)]·CH_2_Cl_2_	*Z* = 2
*M**_r_* = 956.72	*F*(000) = 992
Triclinic, *P*1	*D*_x_ = 1.357 Mg m^−^^3^
*a* = 12.1749 (12) Å	Mo *K*α radiation, λ = 0.71073 Å
*b* = 13.4571 (13) Å	Cell parameters from 8531 reflections
*c* = 15.0439 (15) Å	θ = 2.3–28.3°
α = 107.450 (2)°	µ = 0.49 mm^−^^1^
β = 94.800 (2)°	*T* = 100 K
γ = 90.987 (2)°	Needle, purple
*V* = 2340.8 (4) Å^3^	0.54 × 0.15 × 0.08 mm

### Data collection

**Table:**

Bruker APEX CCD diffractometer	8649 reflections with *I* > 2σ(*I*)
φ and ω scans	*R*_int_ = 0.044
Absorption correction: multi-scan (*SADABS*; Bruker, 2002)	θ_max_ = 28.4°, θ_min_ = 1.4°
*T*_min_ = 0.779, *T*_max_ = 0.962	*h* = −16→16
43417 measured reflections	*k* = −17→17
11596 independent reflections	*l* = −20→20

### Refinement

**Table:**

Refinement on *F*^2^	Primary atom site location: structure-invariant direct methods
Least-squares matrix: full	Secondary atom site location: difference Fourier map
*R*[*F*^2^ > 2σ(*F*^2^)] = 0.056	Hydrogen site location: mixed
*wR*(*F*^2^) = 0.173	H-atom parameters constrained
*S* = 1.00	*w* = 1/[σ^2^(*F*_o_^2^) + (0.117*P*)^2^] where *P* = (*F*_o_^2^ + 2*F*_c_^2^)/3
11596 reflections	(Δ/σ)_max_ = 0.001
632 parameters	Δρ_max_ = 1.12 e Å^−^^3^
353 restraints	Δρ_min_ = −0.44 e Å^−^^3^

### Fractional atomic coordinates and isotropic or equivalent isotropic displacement parameters (Å^2^)

**Table:**

	*x*	*y*	*z*	*U*_iso_*/*U*_eq_	Occ. (<1)
Fe1	0.27912 (2)	0.33446 (2)	0.73638 (2)	0.01878 (10)	
O1	0.28119 (13)	0.22468 (12)	0.55162 (10)	0.0307 (3)	
N1	0.32161 (14)	0.19079 (13)	0.73360 (11)	0.0206 (3)	
N2	0.42828 (13)	0.36153 (12)	0.69803 (11)	0.0187 (3)	
N3	0.23961 (13)	0.48146 (13)	0.74402 (11)	0.0191 (3)	
N4	0.13367 (14)	0.31196 (13)	0.78213 (11)	0.0212 (3)	
N5	0.33989 (13)	0.38736 (13)	0.87552 (12)	0.0213 (3)	
N6	0.35960 (14)	0.48117 (14)	1.02497 (12)	0.0231 (4)	
N7	0.22530 (14)	0.28062 (13)	0.61269 (12)	0.0249 (4)	
C1	0.25682 (18)	0.11657 (15)	0.75314 (15)	0.0252 (4)	
C2	0.31521 (18)	0.02104 (16)	0.73937 (16)	0.0274 (5)	
H2	0.2887	−0.0417	0.7483	0.033*	
C3	0.41421 (18)	0.03794 (16)	0.71148 (15)	0.0260 (4)	
H3	0.4707	−0.0106	0.6973	0.031*	
C4	0.41839 (17)	0.14389 (15)	0.70712 (13)	0.0208 (4)	
C5	0.50755 (16)	0.18962 (15)	0.67927 (13)	0.0198 (4)	
C6	0.51086 (16)	0.29226 (15)	0.67560 (13)	0.0190 (4)	
C7	0.60498 (17)	0.34018 (15)	0.64984 (14)	0.0216 (4)	
H7	0.6720	0.3084	0.6318	0.026*	
C8	0.57892 (16)	0.43990 (15)	0.65640 (13)	0.0204 (4)	
H8	0.6244	0.4912	0.6438	0.024*	
C9	0.46861 (16)	0.45290 (15)	0.68625 (13)	0.0179 (4)	
C10	0.41329 (16)	0.54554 (14)	0.70135 (13)	0.0180 (4)	
C11	0.30580 (16)	0.55733 (15)	0.72738 (13)	0.0194 (4)	
C12	0.24626 (17)	0.65065 (16)	0.73692 (15)	0.0247 (4)	
H12	0.2728	0.7132	0.7275	0.030*	
C13	0.14504 (17)	0.63272 (16)	0.76183 (15)	0.0246 (4)	
H13	0.0876	0.6805	0.7739	0.029*	
C14	0.14132 (16)	0.52730 (15)	0.76648 (13)	0.0207 (4)	
C15	0.05222 (16)	0.48178 (16)	0.79508 (13)	0.0214 (4)	
C16	0.05021 (16)	0.38091 (16)	0.80217 (14)	0.0221 (4)	
C17	−0.04337 (18)	0.33265 (17)	0.82872 (16)	0.0306 (5)	
H17	−0.1110	0.3639	0.8457	0.037*	
C18	−0.01645 (19)	0.23438 (17)	0.82475 (17)	0.0330 (5)	
H18	−0.0617	0.1835	0.8383	0.040*	
C19	0.09441 (17)	0.22134 (16)	0.79584 (15)	0.0251 (4)	
C20	0.15014 (17)	0.12930 (16)	0.78117 (15)	0.0280 (5)	
C21	0.60513 (16)	0.12444 (15)	0.65022 (14)	0.0218 (4)	
C22	0.67985 (18)	0.10442 (16)	0.71722 (15)	0.0278 (5)	
H22	0.6699	0.1330	0.7818	0.033*	
C23	0.76924 (18)	0.04261 (17)	0.69030 (17)	0.0304 (5)	
H23	0.8199	0.0294	0.7366	0.036*	
C24	0.78457 (18)	0.00025 (17)	0.59626 (17)	0.0314 (5)	
H24	0.8454	−0.0420	0.5780	0.038*	
C25	0.71019 (19)	0.02015 (17)	0.52882 (16)	0.0297 (5)	
H25	0.7202	−0.0087	0.4643	0.036*	
C26	0.62130 (18)	0.08223 (16)	0.55575 (15)	0.0258 (4)	
H26	0.5712	0.0960	0.5094	0.031*	
C27	0.47130 (16)	0.63681 (14)	0.68413 (14)	0.0197 (4)	
C28	0.51245 (18)	0.72280 (16)	0.75862 (15)	0.0252 (4)	
H28	0.5013	0.7253	0.8210	0.030*	
C29	0.57002 (19)	0.80518 (17)	0.74160 (17)	0.0305 (5)	
H29	0.5985	0.8631	0.7925	0.037*	
C30	0.58573 (18)	0.80257 (16)	0.65035 (17)	0.0298 (5)	
H30	0.6255	0.8581	0.6388	0.036*	
C31	0.54309 (19)	0.71878 (18)	0.57686 (16)	0.0310 (5)	
H31	0.5529	0.7169	0.5144	0.037*	
C32	0.48549 (18)	0.63668 (16)	0.59382 (15)	0.0267 (4)	
H32	0.4556	0.5798	0.5425	0.032*	
C33	−0.04463 (16)	0.54815 (16)	0.82277 (14)	0.0233 (4)	
C34	−0.05881 (18)	0.59595 (17)	0.91690 (15)	0.0272 (4)	
H34	−0.0092	0.5831	0.9640	0.033*	
C35	−0.14542 (18)	0.66269 (17)	0.94277 (16)	0.0296 (5)	
H35	−0.1544	0.6947	1.0071	0.035*	
C36	−0.21769 (17)	0.68183 (16)	0.87467 (16)	0.0287 (5)	
H36	−0.2754	0.7283	0.8921	0.034*	
C37	−0.20617 (17)	0.63337 (17)	0.78090 (16)	0.0285 (5)	
H37	−0.2570	0.6457	0.7343	0.034*	
C38	−0.12001 (17)	0.56641 (17)	0.75463 (15)	0.0269 (4)	
H38	−0.1127	0.5332	0.6902	0.032*	
C39	0.08236 (15)	0.03490 (17)	0.78418 (19)	0.0339 (6)	0.710 (3)
C40	0.0490 (2)	0.0299 (2)	0.8685 (2)	0.0446 (6)	0.710 (3)
H40	0.0687	0.0854	0.9241	0.054*	0.710 (3)
C41	−0.0130 (3)	−0.0561 (2)	0.8718 (2)	0.0488 (7)	0.710 (3)
H41	−0.0361	−0.0592	0.9297	0.059*	0.710 (3)
C42	−0.0411 (3)	−0.1370 (2)	0.7912 (2)	0.0431 (7)	0.710 (3)
H42	−0.0829	−0.1961	0.7938	0.052*	0.710 (3)
C43	−0.0086 (2)	−0.1324 (2)	0.7066 (2)	0.0386 (6)	0.710 (3)
H43	−0.0288	−0.1879	0.6510	0.046*	0.710 (3)
C44	0.0535 (2)	−0.04654 (19)	0.7032 (2)	0.0327 (6)	0.710 (3)
H44	0.0764	−0.0435	0.6452	0.039*	0.710 (3)
C39'	0.1044 (3)	0.0379 (3)	0.8102 (4)	0.0386 (8)	0.290 (3)
C40'	0.1286 (5)	0.0217 (4)	0.8966 (4)	0.0430 (8)	0.290 (3)
H40'	0.1770	0.0698	0.9431	0.052*	0.290 (3)
C41'	0.0830 (6)	−0.0638 (5)	0.9157 (4)	0.0474 (9)	0.290 (3)
H41'	0.1013	−0.0749	0.9746	0.057*	0.290 (3)
C42'	0.0109 (5)	−0.1329 (4)	0.8489 (5)	0.0452 (9)	0.290 (3)
H42'	−0.0226	−0.1902	0.8627	0.054*	0.290 (3)
C43'	−0.0128 (5)	−0.1189 (5)	0.7619 (5)	0.0414 (8)	0.290 (3)
H43'	−0.0609	−0.1674	0.7154	0.050*	0.290 (3)
C44'	0.0340 (5)	−0.0336 (5)	0.7431 (4)	0.0393 (7)	0.290 (3)
H44'	0.0177	−0.0240	0.6834	0.047*	0.290 (3)
C45	0.31048 (16)	0.47243 (16)	0.93911 (14)	0.0224 (4)	
H45	0.2610	0.5210	0.9257	0.027*	
C46	0.42338 (17)	0.39635 (17)	1.01665 (15)	0.0257 (4)	
H46	0.4677	0.3807	1.0654	0.031*	
C47	0.41053 (16)	0.33881 (16)	0.92439 (14)	0.0240 (4)	
H47	0.4449	0.2752	0.8979	0.029*	
C48	0.34210 (18)	0.56187 (18)	1.11202 (15)	0.0293 (5)	
H48A	0.3083	0.6213	1.0974	0.044*	
H48B	0.4131	0.5848	1.1491	0.044*	
H48C	0.2932	0.5335	1.1479	0.044*	
C49	0.11486 (18)	0.29409 (18)	0.57262 (15)	0.0296 (5)	
C50	0.1022 (2)	0.36033 (19)	0.51683 (16)	0.0344 (5)	
C51	−0.0039 (2)	0.3676 (2)	0.4774 (2)	0.0464 (7)	
H51	−0.0151	0.4120	0.4391	0.056*	
C52	−0.0943 (2)	0.3112 (2)	0.4925 (2)	0.0501 (7)	
H52	−0.1659	0.3190	0.4658	0.060*	
C53	−0.0798 (2)	0.2441 (2)	0.54629 (19)	0.0438 (6)	
H53	−0.1409	0.2050	0.5560	0.053*	
C54	0.0256 (2)	0.23475 (19)	0.58583 (17)	0.0350 (5)	
H54	0.0369	0.1880	0.6218	0.042*	
C55	0.2007 (2)	0.4181 (2)	0.49696 (18)	0.0395 (6)	
H55A	0.2423	0.3694	0.4511	0.059*	
H55B	0.2484	0.4483	0.5551	0.059*	
H55C	0.1750	0.4738	0.4719	0.059*	

### Atomic displacement parameters (Å^2^)

**Table:**

	*U*^11^	*U*^22^	*U*^33^	*U*^12^	*U*^13^	*U*^23^
Fe1	0.02012 (16)	0.01441 (15)	0.02301 (16)	−0.00056 (11)	0.00536 (11)	0.00665 (11)
O1	0.0399 (9)	0.0239 (8)	0.0262 (7)	0.0005 (7)	0.0085 (7)	0.0030 (6)
N1	0.0235 (8)	0.0148 (8)	0.0236 (8)	−0.0022 (6)	0.0039 (7)	0.0057 (6)
N2	0.0221 (8)	0.0136 (7)	0.0201 (8)	0.0002 (6)	0.0014 (6)	0.0047 (6)
N3	0.0195 (8)	0.0184 (8)	0.0203 (8)	0.0002 (6)	0.0026 (6)	0.0070 (6)
N4	0.0241 (8)	0.0170 (8)	0.0210 (8)	−0.0035 (6)	0.0020 (7)	0.0039 (6)
N5	0.0200 (8)	0.0183 (8)	0.0275 (9)	−0.0019 (6)	0.0051 (7)	0.0091 (7)
N6	0.0206 (8)	0.0251 (9)	0.0234 (8)	−0.0040 (7)	0.0029 (7)	0.0073 (7)
N7	0.0256 (9)	0.0180 (8)	0.0302 (9)	−0.0028 (7)	0.0065 (7)	0.0051 (7)
C1	0.0319 (11)	0.0155 (9)	0.0283 (10)	−0.0027 (8)	0.0071 (9)	0.0059 (8)
C2	0.0339 (12)	0.0158 (10)	0.0353 (11)	−0.0008 (8)	0.0058 (9)	0.0112 (9)
C3	0.0306 (11)	0.0144 (9)	0.0332 (11)	−0.0001 (8)	0.0018 (9)	0.0079 (8)
C4	0.0265 (10)	0.0140 (9)	0.0207 (9)	−0.0011 (7)	0.0008 (8)	0.0038 (7)
C5	0.0226 (9)	0.0149 (9)	0.0201 (9)	0.0000 (7)	0.0000 (7)	0.0029 (7)
C6	0.0205 (9)	0.0167 (9)	0.0197 (9)	−0.0004 (7)	0.0015 (7)	0.0053 (7)
C7	0.0222 (10)	0.0167 (9)	0.0254 (10)	−0.0007 (7)	0.0051 (8)	0.0050 (8)
C8	0.0202 (9)	0.0172 (9)	0.0235 (9)	−0.0010 (7)	0.0037 (8)	0.0052 (8)
C9	0.0203 (9)	0.0157 (9)	0.0172 (8)	−0.0022 (7)	0.0010 (7)	0.0043 (7)
C10	0.0218 (9)	0.0148 (9)	0.0172 (8)	−0.0015 (7)	0.0020 (7)	0.0045 (7)
C11	0.0238 (10)	0.0168 (9)	0.0180 (9)	0.0007 (7)	0.0021 (7)	0.0056 (7)
C12	0.0248 (10)	0.0211 (10)	0.0305 (10)	0.0027 (8)	0.0057 (8)	0.0105 (8)
C13	0.0253 (10)	0.0214 (10)	0.0299 (10)	0.0056 (8)	0.0053 (8)	0.0110 (8)
C14	0.0217 (10)	0.0192 (9)	0.0215 (9)	0.0028 (7)	0.0000 (8)	0.0073 (8)
C15	0.0180 (9)	0.0242 (10)	0.0194 (9)	0.0020 (8)	0.0005 (7)	0.0031 (8)
C16	0.0186 (9)	0.0225 (10)	0.0222 (9)	−0.0027 (8)	0.0027 (8)	0.0023 (8)
C17	0.0244 (11)	0.0263 (11)	0.0369 (12)	−0.0063 (9)	0.0106 (9)	0.0016 (9)
C18	0.0296 (11)	0.0222 (11)	0.0431 (13)	−0.0070 (9)	0.0171 (10)	0.0002 (9)
C19	0.0263 (10)	0.0190 (10)	0.0280 (10)	−0.0070 (8)	0.0075 (8)	0.0033 (8)
C20	0.0361 (12)	0.0168 (10)	0.0299 (11)	−0.0071 (9)	0.0098 (9)	0.0040 (8)
C21	0.0222 (10)	0.0125 (9)	0.0303 (10)	−0.0010 (7)	0.0027 (8)	0.0061 (8)
C22	0.0293 (11)	0.0213 (10)	0.0295 (11)	0.0019 (8)	−0.0021 (9)	0.0040 (8)
C23	0.0257 (11)	0.0215 (10)	0.0401 (12)	−0.0002 (8)	−0.0058 (9)	0.0060 (9)
C24	0.0241 (11)	0.0203 (10)	0.0462 (13)	0.0039 (8)	0.0046 (10)	0.0040 (10)
C25	0.0317 (11)	0.0222 (11)	0.0326 (11)	0.0030 (9)	0.0065 (9)	0.0032 (9)
C26	0.0272 (11)	0.0208 (10)	0.0284 (10)	0.0014 (8)	0.0018 (9)	0.0060 (8)
C27	0.0208 (9)	0.0135 (9)	0.0261 (10)	0.0011 (7)	0.0047 (8)	0.0071 (7)
C28	0.0324 (11)	0.0179 (10)	0.0266 (10)	0.0003 (8)	0.0008 (9)	0.0091 (8)
C29	0.0333 (12)	0.0163 (10)	0.0396 (12)	−0.0045 (9)	−0.0050 (10)	0.0077 (9)
C30	0.0291 (11)	0.0172 (10)	0.0465 (13)	−0.0027 (8)	0.0046 (10)	0.0147 (9)
C31	0.0384 (13)	0.0277 (11)	0.0317 (11)	−0.0008 (9)	0.0102 (10)	0.0145 (9)
C32	0.0345 (11)	0.0190 (10)	0.0256 (10)	−0.0049 (8)	0.0055 (9)	0.0047 (8)
C33	0.0200 (9)	0.0225 (10)	0.0262 (10)	−0.0005 (8)	0.0036 (8)	0.0051 (8)
C34	0.0269 (11)	0.0274 (11)	0.0259 (10)	0.0007 (9)	0.0042 (9)	0.0053 (9)
C35	0.0277 (11)	0.0270 (11)	0.0304 (11)	−0.0017 (9)	0.0106 (9)	0.0012 (9)
C36	0.0219 (10)	0.0204 (10)	0.0443 (13)	0.0012 (8)	0.0107 (9)	0.0085 (9)
C37	0.0210 (10)	0.0266 (11)	0.0371 (12)	0.0006 (8)	0.0003 (9)	0.0089 (9)
C38	0.0241 (10)	0.0287 (11)	0.0261 (10)	0.0025 (8)	0.0025 (8)	0.0053 (9)
C39	0.0362 (12)	0.0202 (10)	0.0475 (14)	−0.0028 (10)	0.0134 (11)	0.0115 (10)
C40	0.0508 (13)	0.0324 (11)	0.0524 (13)	−0.0122 (11)	0.0136 (12)	0.0136 (11)
C41	0.0549 (13)	0.0363 (12)	0.0594 (14)	−0.0140 (11)	0.0163 (12)	0.0188 (11)
C42	0.0425 (13)	0.0271 (11)	0.0641 (15)	−0.0055 (11)	0.0141 (12)	0.0184 (11)
C43	0.0350 (13)	0.0198 (11)	0.0596 (15)	−0.0010 (10)	0.0105 (12)	0.0088 (12)
C44	0.0293 (12)	0.0187 (10)	0.0510 (14)	−0.0003 (9)	0.0112 (11)	0.0098 (10)
C39'	0.0400 (15)	0.0239 (14)	0.0525 (16)	−0.0055 (13)	0.0152 (14)	0.0101 (13)
C40'	0.0490 (16)	0.0297 (14)	0.0531 (16)	−0.0082 (14)	0.0167 (15)	0.0139 (14)
C41'	0.0541 (17)	0.0339 (16)	0.0570 (17)	−0.0093 (16)	0.0170 (16)	0.0154 (15)
C42'	0.0477 (17)	0.0308 (16)	0.0609 (18)	−0.0076 (15)	0.0172 (16)	0.0169 (15)
C43'	0.0413 (15)	0.0258 (13)	0.0603 (16)	−0.0052 (12)	0.0136 (14)	0.0156 (14)
C44'	0.0388 (13)	0.0240 (12)	0.0568 (15)	−0.0051 (12)	0.0115 (13)	0.0133 (12)
C45	0.0204 (9)	0.0237 (10)	0.0246 (10)	−0.0008 (8)	0.0037 (8)	0.0093 (8)
C46	0.0225 (10)	0.0301 (11)	0.0282 (10)	−0.0010 (8)	0.0022 (8)	0.0146 (9)
C47	0.0214 (10)	0.0231 (10)	0.0303 (11)	0.0012 (8)	0.0059 (8)	0.0116 (9)
C48	0.0293 (11)	0.0315 (12)	0.0242 (10)	−0.0047 (9)	0.0036 (9)	0.0042 (9)
C49	0.0302 (11)	0.0294 (11)	0.0261 (10)	−0.0020 (9)	−0.0002 (9)	0.0043 (9)
C50	0.0375 (13)	0.0360 (13)	0.0301 (11)	−0.0008 (10)	−0.0006 (10)	0.0117 (10)
C51	0.0398 (14)	0.0577 (17)	0.0471 (15)	−0.0045 (13)	−0.0084 (12)	0.0275 (14)
C52	0.0325 (14)	0.0608 (19)	0.0584 (18)	−0.0022 (13)	−0.0091 (12)	0.0238 (15)
C53	0.0361 (14)	0.0482 (16)	0.0430 (14)	−0.0152 (12)	−0.0041 (11)	0.0108 (12)
C54	0.0352 (12)	0.0334 (13)	0.0340 (12)	−0.0082 (10)	0.0006 (10)	0.0077 (10)
C55	0.0421 (14)	0.0446 (15)	0.0370 (13)	−0.0018 (11)	−0.0013 (11)	0.0220 (11)

### Geometric parameters (Å, º)

**Table:**

Fe1—N7	1.8406 (18)	C27—C32	1.383 (3)
Fe1—N1	1.9992 (17)	C27—C28	1.398 (3)
Fe1—N4	2.0030 (17)	C28—C29	1.400 (3)
Fe1—N2	2.0105 (16)	C28—H28	0.9500
Fe1—N3	2.0159 (16)	C29—C30	1.392 (3)
Fe1—N5	2.0651 (17)	C29—H29	0.9500
O1—N7	1.257 (2)	C30—C31	1.378 (3)
N1—C1	1.376 (2)	C30—H30	0.9500
N1—C4	1.378 (3)	C31—C32	1.396 (3)
N2—C6	1.376 (2)	C31—H31	0.9500
N2—C9	1.382 (2)	C32—H32	0.9500
N3—C14	1.377 (2)	C33—C34	1.395 (3)
N3—C11	1.383 (2)	C33—C38	1.398 (3)
N4—C16	1.379 (3)	C34—C35	1.401 (3)
N4—C19	1.380 (3)	C34—H34	0.9500
N5—C45	1.330 (3)	C35—C36	1.380 (3)
N5—C47	1.381 (3)	C35—H35	0.9500
N6—C45	1.348 (3)	C36—C37	1.385 (3)
N6—C46	1.372 (3)	C36—H36	0.9500
N6—C48	1.463 (3)	C37—C38	1.399 (3)
N7—C49	1.464 (3)	C37—H37	0.9500
C1—C20	1.396 (3)	C38—H38	0.9500
C1—C2	1.448 (3)	C39—C40	1.383 (3)
C2—C3	1.346 (3)	C39—C44	1.387 (3)
C2—H2	0.9500	C40—C41	1.387 (3)
C3—C4	1.447 (3)	C40—H40	0.9500
C3—H3	0.9500	C41—C42	1.378 (3)
C4—C5	1.393 (3)	C41—H41	0.9500
C5—C6	1.399 (3)	C42—C43	1.382 (3)
C5—C21	1.505 (3)	C42—H42	0.9500
C6—C7	1.441 (3)	C43—C44	1.386 (3)
C7—C8	1.361 (3)	C43—H43	0.9500
C7—H7	0.9500	C44—H44	0.9500
C8—C9	1.448 (3)	C39'—C44'	1.387 (3)
C8—H8	0.9500	C39'—C40'	1.389 (3)
C9—C10	1.394 (3)	C40'—C41'	1.385 (3)
C10—C11	1.395 (3)	C40'—H40'	0.9500
C10—C27	1.505 (3)	C41'—C42'	1.383 (3)
C11—C12	1.437 (3)	C41'—H41'	0.9500
C12—C13	1.356 (3)	C42'—C43'	1.385 (3)
C12—H12	0.9500	C42'—H42'	0.9500
C13—C14	1.441 (3)	C43'—C44'	1.386 (3)
C13—H13	0.9500	C43'—H43'	0.9500
C14—C15	1.396 (3)	C44'—H44'	0.9500
C15—C16	1.393 (3)	C45—H45	0.9500
C15—C33	1.502 (3)	C46—C47	1.366 (3)
C16—C17	1.444 (3)	C46—H46	0.9500
C17—C18	1.353 (3)	C47—H47	0.9500
C17—H17	0.9500	C48—H48A	0.9800
C18—C19	1.450 (3)	C48—H48B	0.9800
C18—H18	0.9500	C48—H48C	0.9800
C19—C20	1.390 (3)	C49—C50	1.398 (3)
C20—C39	1.517 (3)	C49—C54	1.400 (3)
C20—C39'	1.534 (4)	C50—C51	1.392 (3)
C21—C22	1.391 (3)	C50—C55	1.516 (3)
C21—C26	1.396 (3)	C51—C52	1.398 (4)
C22—C23	1.394 (3)	C51—H51	0.9500
C22—H22	0.9500	C52—C53	1.387 (4)
C23—C24	1.388 (3)	C52—H52	0.9500
C23—H23	0.9500	C53—C54	1.392 (4)
C24—C25	1.392 (3)	C53—H53	0.9500
C24—H24	0.9500	C54—H54	0.9500
C25—C26	1.391 (3)	C55—H55A	0.9800
C25—H25	0.9500	C55—H55B	0.9800
C26—H26	0.9500	C55—H55C	0.9800
			
N7—Fe1—N1	88.22 (7)	C25—C26—H26	119.7
N7—Fe1—N4	92.86 (7)	C21—C26—H26	119.7
N1—Fe1—N4	90.81 (7)	C32—C27—C28	118.70 (18)
N7—Fe1—N2	90.38 (7)	C32—C27—C10	120.36 (18)
N1—Fe1—N2	89.75 (7)	C28—C27—C10	120.94 (17)
N4—Fe1—N2	176.73 (6)	C27—C28—C29	120.2 (2)
N7—Fe1—N3	93.95 (7)	C27—C28—H28	119.9
N1—Fe1—N3	177.80 (7)	C29—C28—H28	119.9
N4—Fe1—N3	89.48 (7)	C30—C29—C28	120.3 (2)
N2—Fe1—N3	89.84 (7)	C30—C29—H29	119.9
N7—Fe1—N5	177.15 (7)	C28—C29—H29	119.9
N1—Fe1—N5	89.01 (7)	C31—C30—C29	119.5 (2)
N4—Fe1—N5	86.52 (6)	C31—C30—H30	120.3
N2—Fe1—N5	90.27 (6)	C29—C30—H30	120.3
N3—Fe1—N5	88.83 (7)	C30—C31—C32	120.3 (2)
C1—N1—C4	106.02 (16)	C30—C31—H31	119.9
C1—N1—Fe1	126.48 (14)	C32—C31—H31	119.9
C4—N1—Fe1	127.41 (13)	C27—C32—C31	121.0 (2)
C6—N2—C9	105.32 (16)	C27—C32—H32	119.5
C6—N2—Fe1	127.36 (13)	C31—C32—H32	119.5
C9—N2—Fe1	127.29 (13)	C34—C33—C38	118.70 (19)
C14—N3—C11	105.59 (16)	C34—C33—C15	120.68 (18)
C14—N3—Fe1	127.36 (13)	C38—C33—C15	120.57 (18)
C11—N3—Fe1	127.05 (13)	C33—C34—C35	120.8 (2)
C16—N4—C19	105.71 (17)	C33—C34—H34	119.6
C16—N4—Fe1	127.68 (14)	C35—C34—H34	119.6
C19—N4—Fe1	126.55 (14)	C36—C35—C34	119.9 (2)
C45—N5—C47	105.31 (17)	C36—C35—H35	120.1
C45—N5—Fe1	126.23 (14)	C34—C35—H35	120.1
C47—N5—Fe1	128.22 (14)	C35—C36—C37	120.1 (2)
C45—N6—C46	107.23 (17)	C35—C36—H36	119.9
C45—N6—C48	126.44 (18)	C37—C36—H36	119.9
C46—N6—C48	126.20 (18)	C36—C37—C38	120.2 (2)
O1—N7—C49	111.42 (17)	C36—C37—H37	119.9
O1—N7—Fe1	122.54 (14)	C38—C37—H37	119.9
C49—N7—Fe1	126.03 (14)	C33—C38—C37	120.3 (2)
N1—C1—C20	125.95 (19)	C33—C38—H38	119.9
N1—C1—C2	109.86 (18)	C37—C38—H38	119.9
C20—C1—C2	124.18 (19)	C40—C39—C44	119.6 (2)
C3—C2—C1	107.11 (18)	C40—C39—C20	119.7 (2)
C3—C2—H2	126.4	C44—C39—C20	120.6 (2)
C1—C2—H2	126.4	C39—C40—C41	120.1 (2)
C2—C3—C4	107.19 (19)	C39—C40—H40	119.9
C2—C3—H3	126.4	C41—C40—H40	119.9
C4—C3—H3	126.4	C42—C41—C40	120.0 (2)
N1—C4—C5	126.01 (18)	C42—C41—H41	120.0
N1—C4—C3	109.82 (17)	C40—C41—H41	120.0
C5—C4—C3	124.17 (19)	C41—C42—C43	120.2 (2)
C4—C5—C6	123.69 (18)	C41—C42—H42	119.9
C4—C5—C21	118.14 (17)	C43—C42—H42	119.9
C6—C5—C21	118.17 (17)	C42—C43—C44	119.8 (2)
N2—C6—C5	125.63 (18)	C42—C43—H43	120.1
N2—C6—C7	111.00 (16)	C44—C43—H43	120.1
C5—C6—C7	123.34 (18)	C43—C44—C39	120.2 (2)
C8—C7—C6	106.51 (17)	C43—C44—H44	119.9
C8—C7—H7	126.7	C39—C44—H44	119.9
C6—C7—H7	126.7	C44'—C39'—C40'	118.7 (3)
C7—C8—C9	106.94 (17)	C44'—C39'—C20	115.9 (4)
C7—C8—H8	126.5	C40'—C39'—C20	125.4 (4)
C9—C8—H8	126.5	C41'—C40'—C39'	120.8 (3)
N2—C9—C10	125.88 (17)	C41'—C40'—H40'	119.6
N2—C9—C8	110.22 (16)	C39'—C40'—H40'	119.6
C10—C9—C8	123.90 (17)	C42'—C41'—C40'	119.7 (3)
C9—C10—C11	124.00 (18)	C42'—C41'—H41'	120.1
C9—C10—C27	117.87 (17)	C40'—C41'—H41'	120.1
C11—C10—C27	118.07 (17)	C41'—C42'—C43'	120.2 (3)
N3—C11—C10	125.90 (17)	C41'—C42'—H42'	119.9
N3—C11—C12	110.10 (17)	C43'—C42'—H42'	119.9
C10—C11—C12	123.95 (18)	C42'—C43'—C44'	119.6 (3)
C13—C12—C11	107.15 (18)	C42'—C43'—H43'	120.2
C13—C12—H12	126.4	C44'—C43'—H43'	120.2
C11—C12—H12	126.4	C43'—C44'—C39'	120.9 (3)
C12—C13—C14	106.88 (18)	C43'—C44'—H44'	119.5
C12—C13—H13	126.6	C39'—C44'—H44'	119.5
C14—C13—H13	126.6	N5—C45—N6	111.56 (18)
N3—C14—C15	125.67 (18)	N5—C45—H45	124.2
N3—C14—C13	110.25 (17)	N6—C45—H45	124.2
C15—C14—C13	123.99 (18)	C47—C46—N6	106.35 (18)
C16—C15—C14	123.85 (18)	C47—C46—H46	126.8
C16—C15—C33	118.98 (18)	N6—C46—H46	126.8
C14—C15—C33	117.14 (18)	C46—C47—N5	109.54 (18)
N4—C16—C15	125.80 (18)	C46—C47—H47	125.2
N4—C16—C17	110.35 (18)	N5—C47—H47	125.2
C15—C16—C17	123.81 (19)	N6—C48—H48A	109.5
C18—C17—C16	106.94 (19)	N6—C48—H48B	109.5
C18—C17—H17	126.5	H48A—C48—H48B	109.5
C16—C17—H17	126.5	N6—C48—H48C	109.5
C17—C18—C19	107.05 (19)	H48A—C48—H48C	109.5
C17—C18—H18	126.5	H48B—C48—H48C	109.5
C19—C18—H18	126.5	C50—C49—C54	121.5 (2)
N4—C19—C20	125.71 (19)	C50—C49—N7	119.3 (2)
N4—C19—C18	109.94 (19)	C54—C49—N7	119.0 (2)
C20—C19—C18	124.31 (19)	C51—C50—C49	117.2 (2)
C19—C20—C1	124.37 (19)	C51—C50—C55	121.6 (2)
C19—C20—C39	115.73 (15)	C49—C50—C55	121.2 (2)
C1—C20—C39	119.39 (15)	C50—C51—C52	121.8 (2)
C19—C20—C39'	120.97 (16)	C50—C51—H51	119.1
C1—C20—C39'	113.99 (16)	C52—C51—H51	119.1
C22—C21—C26	119.05 (19)	C53—C52—C51	120.2 (2)
C22—C21—C5	120.38 (18)	C53—C52—H52	119.9
C26—C21—C5	120.57 (18)	C51—C52—H52	119.9
C21—C22—C23	120.4 (2)	C52—C53—C54	119.1 (2)
C21—C22—H22	119.8	C52—C53—H53	120.5
C23—C22—H22	119.8	C54—C53—H53	120.5
C24—C23—C22	120.3 (2)	C53—C54—C49	120.1 (2)
C24—C23—H23	119.8	C53—C54—H54	119.9
C22—C23—H23	119.8	C49—C54—H54	119.9
C23—C24—C25	119.6 (2)	C50—C55—H55A	109.5
C23—C24—H24	120.2	C50—C55—H55B	109.5
C25—C24—H24	120.2	H55A—C55—H55B	109.5
C26—C25—C24	120.1 (2)	C50—C55—H55C	109.5
C26—C25—H25	120.0	H55A—C55—H55C	109.5
C24—C25—H25	120.0	H55B—C55—H55C	109.5
C25—C26—C21	120.6 (2)		
			
N1—Fe1—N7—O1	−50.99 (16)	N1—C1—C20—C39	169.7 (2)
N4—Fe1—N7—O1	−141.71 (16)	C2—C1—C20—C39	−9.6 (3)
N2—Fe1—N7—O1	38.74 (16)	N1—C1—C20—C39'	−172.4 (3)
N3—Fe1—N7—O1	128.61 (16)	C2—C1—C20—C39'	8.3 (4)
N1—Fe1—N7—C49	127.69 (17)	C4—C5—C21—C22	−76.4 (2)
N4—Fe1—N7—C49	36.97 (17)	C6—C5—C21—C22	104.5 (2)
N2—Fe1—N7—C49	−142.57 (17)	C4—C5—C21—C26	102.9 (2)
N3—Fe1—N7—C49	−52.70 (17)	C6—C5—C21—C26	−76.3 (2)
C4—N1—C1—C20	−178.8 (2)	C26—C21—C22—C23	−0.3 (3)
Fe1—N1—C1—C20	−1.9 (3)	C5—C21—C22—C23	178.93 (19)
C4—N1—C1—C2	0.6 (2)	C21—C22—C23—C24	−0.1 (3)
Fe1—N1—C1—C2	177.43 (14)	C22—C23—C24—C25	0.2 (3)
N1—C1—C2—C3	−0.2 (2)	C23—C24—C25—C26	0.1 (3)
C20—C1—C2—C3	179.2 (2)	C24—C25—C26—C21	−0.5 (3)
C1—C2—C3—C4	−0.3 (2)	C22—C21—C26—C25	0.6 (3)
C1—N1—C4—C5	178.72 (19)	C5—C21—C26—C25	−178.62 (19)
Fe1—N1—C4—C5	1.9 (3)	C9—C10—C27—C32	71.9 (2)
C1—N1—C4—C3	−0.8 (2)	C11—C10—C27—C32	−105.4 (2)
Fe1—N1—C4—C3	−177.57 (13)	C9—C10—C27—C28	−107.7 (2)
C2—C3—C4—N1	0.7 (2)	C11—C10—C27—C28	75.1 (2)
C2—C3—C4—C5	−178.82 (19)	C32—C27—C28—C29	−2.1 (3)
N1—C4—C5—C6	0.8 (3)	C10—C27—C28—C29	177.46 (19)
C3—C4—C5—C6	−179.79 (19)	C27—C28—C29—C30	0.7 (3)
N1—C4—C5—C21	−178.29 (18)	C28—C29—C30—C31	0.7 (3)
C3—C4—C5—C21	1.1 (3)	C29—C30—C31—C32	−0.6 (3)
C9—N2—C6—C5	178.61 (18)	C28—C27—C32—C31	2.3 (3)
Fe1—N2—C6—C5	−2.9 (3)	C10—C27—C32—C31	−177.3 (2)
C9—N2—C6—C7	0.4 (2)	C30—C31—C32—C27	−0.9 (3)
Fe1—N2—C6—C7	178.86 (13)	C16—C15—C33—C34	77.4 (3)
C4—C5—C6—N2	−0.3 (3)	C14—C15—C33—C34	−100.5 (2)
C21—C5—C6—N2	178.81 (17)	C16—C15—C33—C38	−105.0 (2)
C4—C5—C6—C7	177.76 (18)	C14—C15—C33—C38	77.1 (3)
C21—C5—C6—C7	−3.1 (3)	C38—C33—C34—C35	−1.3 (3)
N2—C6—C7—C8	−0.2 (2)	C15—C33—C34—C35	176.3 (2)
C5—C6—C7—C8	−178.53 (18)	C33—C34—C35—C36	−0.1 (3)
C6—C7—C8—C9	0.0 (2)	C34—C35—C36—C37	1.4 (3)
C6—N2—C9—C10	−179.75 (18)	C35—C36—C37—C38	−1.3 (3)
Fe1—N2—C9—C10	1.8 (3)	C34—C33—C38—C37	1.5 (3)
C6—N2—C9—C8	−0.4 (2)	C15—C33—C38—C37	−176.1 (2)
Fe1—N2—C9—C8	−178.86 (12)	C36—C37—C38—C33	−0.2 (3)
C7—C8—C9—N2	0.2 (2)	C19—C20—C39—C40	−70.3 (2)
C7—C8—C9—C10	179.63 (18)	C1—C20—C39—C40	117.5 (2)
N2—C9—C10—C11	−2.7 (3)	C39'—C20—C39—C40	42.0 (5)
C8—C9—C10—C11	177.99 (18)	C19—C20—C39—C44	109.7 (2)
N2—C9—C10—C27	−179.73 (17)	C1—C20—C39—C44	−62.4 (2)
C8—C9—C10—C27	1.0 (3)	C39'—C20—C39—C44	−137.9 (5)
C14—N3—C11—C10	−179.38 (18)	C44—C39—C40—C41	−0.1 (2)
Fe1—N3—C11—C10	0.4 (3)	C20—C39—C40—C41	179.96 (18)
C14—N3—C11—C12	−1.8 (2)	C39—C40—C41—C42	0.3 (4)
Fe1—N3—C11—C12	178.00 (13)	C40—C41—C42—C43	−0.7 (4)
C9—C10—C11—N3	1.6 (3)	C41—C42—C43—C44	0.8 (4)
C27—C10—C11—N3	178.59 (17)	C42—C43—C44—C39	−0.5 (4)
C9—C10—C11—C12	−175.72 (19)	C40—C39—C44—C43	0.2 (3)
C27—C10—C11—C12	1.3 (3)	C20—C39—C44—C43	−179.88 (17)
N3—C11—C12—C13	1.6 (2)	C19—C20—C39'—C44'	89.8 (4)
C10—C11—C12—C13	179.29 (19)	C1—C20—C39'—C44'	−99.1 (3)
C11—C12—C13—C14	−0.8 (2)	C39—C20—C39'—C44'	13.4 (5)
C11—N3—C14—C15	−175.27 (18)	C19—C20—C39'—C40'	−90.7 (3)
Fe1—N3—C14—C15	5.0 (3)	C1—C20—C39'—C40'	80.3 (3)
C11—N3—C14—C13	1.3 (2)	C39—C20—C39'—C40'	−167.1 (5)
Fe1—N3—C14—C13	−178.49 (13)	C44'—C39'—C40'—C41'	−0.4 (3)
C12—C13—C14—N3	−0.3 (2)	C20—C39'—C40'—C41'	−179.8 (2)
C12—C13—C14—C15	176.31 (19)	C39'—C40'—C41'—C42'	−1.2 (5)
N3—C14—C15—C16	−1.9 (3)	C40'—C41'—C42'—C43'	2.3 (6)
C13—C14—C15—C16	−178.01 (19)	C41'—C42'—C43'—C44'	−1.7 (6)
N3—C14—C15—C33	175.84 (18)	C42'—C43'—C44'—C39'	0.0 (6)
C13—C14—C15—C33	−0.3 (3)	C40'—C39'—C44'—C43'	1.0 (4)
C19—N4—C16—C15	−177.98 (19)	C20—C39'—C44'—C43'	−179.5 (3)
Fe1—N4—C16—C15	−0.8 (3)	C47—N5—C45—N6	0.9 (2)
C19—N4—C16—C17	−0.2 (2)	Fe1—N5—C45—N6	175.57 (12)
Fe1—N4—C16—C17	177.06 (14)	C46—N6—C45—N5	−0.7 (2)
C14—C15—C16—N4	−0.3 (3)	C48—N6—C45—N5	−176.69 (18)
C33—C15—C16—N4	−178.01 (18)	C45—N6—C46—C47	0.3 (2)
C14—C15—C16—C17	−177.84 (19)	C48—N6—C46—C47	176.25 (18)
C33—C15—C16—C17	4.5 (3)	N6—C46—C47—N5	0.3 (2)
N4—C16—C17—C18	0.0 (2)	C45—N5—C47—C46	−0.7 (2)
C15—C16—C17—C18	177.9 (2)	Fe1—N5—C47—C46	−175.24 (13)
C16—C17—C18—C19	0.1 (3)	O1—N7—C49—C50	−74.2 (2)
C16—N4—C19—C20	178.1 (2)	Fe1—N7—C49—C50	107.0 (2)
Fe1—N4—C19—C20	0.8 (3)	O1—N7—C49—C54	101.4 (2)
C16—N4—C19—C18	0.2 (2)	Fe1—N7—C49—C54	−77.4 (2)
Fe1—N4—C19—C18	−177.01 (14)	C54—C49—C50—C51	2.1 (4)
C17—C18—C19—N4	−0.2 (3)	N7—C49—C50—C51	177.6 (2)
C17—C18—C19—C20	−178.1 (2)	C54—C49—C50—C55	−175.3 (2)
N4—C19—C20—C1	2.3 (4)	N7—C49—C50—C55	0.2 (3)
C18—C19—C20—C1	179.9 (2)	C49—C50—C51—C52	0.0 (4)
N4—C19—C20—C39	−169.4 (2)	C55—C50—C51—C52	177.4 (3)
C18—C19—C20—C39	8.1 (3)	C50—C51—C52—C53	−1.4 (5)
N4—C19—C20—C39'	172.4 (3)	C51—C52—C53—C54	0.8 (4)
C18—C19—C20—C39'	−10.1 (4)	C52—C53—C54—C49	1.2 (4)
N1—C1—C20—C19	−1.7 (4)	C50—C49—C54—C53	−2.7 (4)
C2—C1—C20—C19	179.0 (2)	N7—C49—C54—C53	−178.3 (2)
